# Indirect Induction Sintering of Metal Parts Produced through Material Extrusion Additive Manufacturing

**DOI:** 10.3390/ma16020885

**Published:** 2023-01-16

**Authors:** Manuel Ortega Varela de Seijas, Andreas Bardenhagen, Thomas Rohr, Enrico Stoll

**Affiliations:** 1 Institute of Aeronautics and Astronautics, Technical University of Berlin, 10587 Berlin, Germany; 2 European Space Agency (ESA), 2201 AA Noordwijk, The Netherlands

**Keywords:** material extrusion, additive manufacturing, sintering, induction, stainless steel 316L

## Abstract

Avoiding loose powders and resins, material extrusion additive manufacturing is a powerful technique to produce near-net shape parts, being a cheap and safe alternative for developing complex industrial-grade products. Filaments embedded with a high packing density of metallic or ceramic granules are being increasingly used, resulting in almost fully dense parts, whereby geometries are shaped, debinded and sintered sequentially until the completion of the part. Traditionally, “brown” debinded geometries are transported to conventional furnaces to densify the powder compacts, requiring careful tailoring of the heating profiles and sintering environment. This approach is decoupled and often involves time-consuming post-processing, whereby after the completion of the shaping and debinding steps, the parts need to be transported to a sintering furnace. Here, it is shown that sintering via indirect induction heating of a highly filled commercially available filament embedded with stainless steel 316L powder can be an effective route to densify Fused Filament Fabricated (FFF) parts. The results show that densities of 99.8% can be reached with very short soaking times, representing a significant improvement compared to prior methods. A hybrid machine is proposed, whereby a custom-built machine is integrated with an induction heater to combine FFF with local indirect induction sintering. Sintering in situ, without the need for part transportation, simplifies the processing of metal parts produced through material extrusion additive manufacturing.

## 1. Introduction

Additive Manufacturing (AM) is a growing field for the production of high-performance near-net functional parts for the aerospace [1,2], automotive [3,4], biomedical [5,6], and energy industries [7], showing the potential to be printed on the Moon [8]. However, the infancy of this technology today means that it is in an early development stage, with multiple parallel techniques being further matured. Depending on the nature of the feedstock, these techniques can be classified as powder, filament, or resin-based techniques [9]. The most common processes for metals include Powder Bed Fusion (PBF), with subvariants Selective Laser Melting (SLM) and Electron Beam Melting (EBM), which employ a laser or an electron beam to repetitively melt 2D slices of a 3D object on a powder bed [10,11,12]. Powder can also be blown into a molten pool, known as Direct Metal Deposition (DMD), where the powder is blown and fused together by means of a laser or an electron beam [13]. On the other hand, electric or plasma Wire Arc Additive Manufacturing (WAAM), Wire Electron Deposition, and Wire Laser Deposition employ metal wires instead of powders, enabling the production of larger structures [14].

A novel filament-based material extrusion AM alternative for shaping parts is Fused Filament Fabrication (FFF), whereby the filament is extruded through a heated nozzle. With the expiration of the technology’s patent, there is now a large open-source development community as well as DIY machines, significantly lowering the costs of this technology. A wide variety of materials can be extruded with low complexity using process parameter adaptations, with the most popular being thermoplastics, such as Acrylonitrile Butadiene Styrene (ABS) [15], PolyLactic Acid (PLA) [16], Thermoplastic PolyUrethane (TPU) [17], aliphatic PolyAmides (PA, or Nylon) [18], high-performance polymers, such as PolyEther Ether Ketone PEEK [19] or PolyEtherimide (PEI) [20], composites [21,22], and more recently, metals and ceramics from the filaments embedded with powdered granules, even showcasing the feasibility of conducting the process with Martian regolith simulants [23]. Compared to other methods for producing metallic structures via AM, the main advantage of FFF is the immobilization of the metallic particles within the polymeric binder matrix, which reduces the hazards of fine metal powders. Furthermore, FFF allows for multi-material metal–ceramic AM [24] and eliminates some common problems of metal AM, such as the accumulation of tensile residual stresses or the complicated and time-consuming adaptation required for new materials [25].

**Figure 1:**
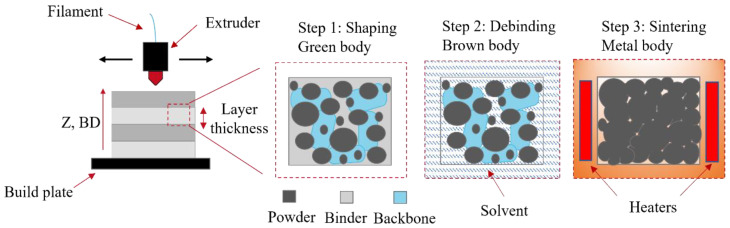
Illustration of the FFF processing steps to produce dense metal or ceramic parts.

In this approach, highly filled thermoplastic filaments embedded with micron-sized metal powders are used to produce dense parts after a debinding and sintering stage, as illustrated in Figure 1, with Z being the build direction (BD). First, the melted filament is deposited on a build platform and shaped layer-by-layer by following a 2D slice of a 3D CAD object until the completion of the “green” part (step 1). Typically, a multi-binder system is used for the formulation of the filament feedstock, which is composed of a combination of polymers, waxes, and additives [25,26], with the debinding stage dependent on the chosen binder system (e.g., with solvents [27,28], catalysts [29], or through pure thermal decomposition [30]). The result of the debinding step, otherwise known as the “brown” part, consists of metal granules and a residual secondary binder or backbone maintaining the shape of the part (step 2). The subsequent sintering step decomposes the backbone from the debinded geometry and causes the metal particles to fuse, resulting in a densified solid mass (step 3). Representative alloys that have been successfully shaped using FFF and subsequently sintered include stainless steel 17-4 PH [31], inconel super alloy IN 718 [32], titanium Ti6Al4V [33], and copper Cu [34]. However, conventional sintering is carried out in sophisticated furnaces that can maintain a tailored neutral atmosphere, being out of reach for most users. Here, we show that cost-effective equipment can be combined with metal FFF AM by using standard off-the-shelf equipment.

The solid-state sintering of powder compacts results from the reduced surface energy of particles in close proximity, whereby mass transport diffusion takes place, with the most common approach performed through solid-state furnace sintering [35]. Other highly effective routes have been developed more recently, such as microwave sintering [36,37], Spark Plasma Sintering (SPS) or Field-Assisted Sintering (FAST) [38], or via direct [39,40,41] or indirect [42,43] induction. An advantage of microwave, SPS/FAST, and induction sintering, in contrast to conventional sintering, is the capability to sinter in only a few minutes rather than hours or days [36,38,41]. Low sintering times are possible with microwaves, SPS, and induction since high heating rates are attained due to the internal heating of the part as opposed to the external heating of conventional furnace sintering. However, the disadvantage of microwave and SPS/FAST sintering lies in the fact that brown bodies have to be transported to expensive and bulky equipment whilst restricting the materials that can be effectively sintered, which need to absorb microwaves or be subjected to high pressures in SPS. On the other hand, induction heating employs cost-effective equipment and can indirectly heat any material with the use of an intermediate conductive susceptor, which absorbs electromagnetic energy and converts it to heat via the joule heating effect. To date, indirect induction sintering has not been reported for parts shaped via FFF.

Pure induction heating is a result of induced eddy currents, which are created by an alternating magnetic field, and thus can only be realized when using conductive materials [44]. Consequently, indirect induction heating is typically used for non-conductive material feedstocks, whereby a conductive susceptor crucible is employed. The powder parameters influencing the net sintered density and microstructure include morphology, powder packing density, alloy composition, and the presence of impurities. High powder volume/packing densities within the feedstock material are desired to decrease the shrinkage of the part after solid-state sintering whilst rendering high densities, with typical volume loadings of 55–70% to ensure the flowability of the feedstock material [45,46]. It is advantageous to employ fine micron-sized powders to increase the packing density within the neighboring particles, ensuring a decrease in interstitial voids and a higher density upon sintering. The main factors to consider when choosing a filament diameter are the resolution of the required part and the flowability of the extruded mass. To avoid clogging the nozzle when using micron-sized metallic powders, a nozzle aperture size of at least 0.4 mm is recommended when using standard high-packing-density commercially available filaments with typical available diameters of 1.75 [47]. The driving parameters for the sintering stage include the temperature, pressure, atmosphere, and heating and cooling rates of the process. Today, all sintering techniques for parts produced through FFF are performed after shaping and debinding the geometry, using dedicated equipment in a decoupled approach. In preparation for coupling a sintering heater within an FFF machine, an initial investigation is presented in this study by considering highly effective sintering routes.

**Figure 2:**
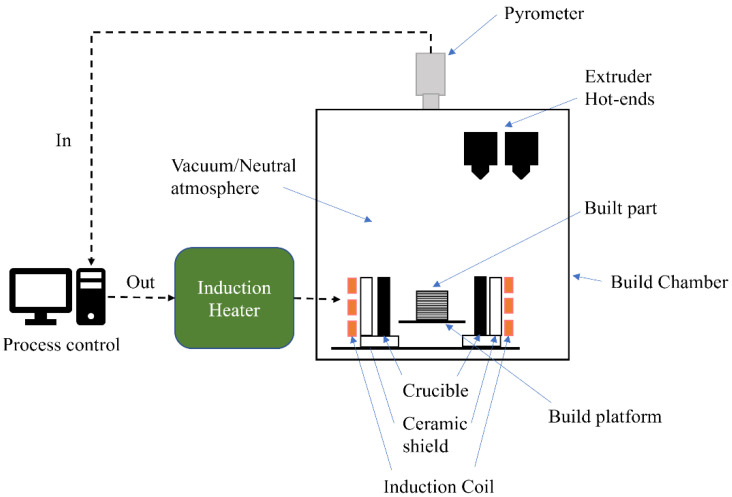
Illustration of the hybrid FFF and indirect induction sintering enabling in situ processing.

In this work, the sintering of a highly filled stainless steel 316 L filament shaped using FFF is examined in combination with an indirect induction heater via a decoupled approach, whereby the parts were first shaped, debinded, and subsequently sintered. The decoupled results indicate that indirect induction sintering can be combined with FFF. A hybrid machine is proposed in Figure 2, integrating a traditional FFF device with an indirect induction sintering treatment to densify metal parts in situ without the need for part transportation. This approach enables focusing the energy effectively along the 3D volume of the shaped part, opening new routes to process parts locally without the use of consumables or separate expensive equipment. The combination of material extrusion AM with local sintering is a novel hybrid additive manufacturing method with a patent application filed by the Technical University of Berlin [48]. The results from the decoupled approach are characterized using optical microscopy, electron backscatter diffraction maps (EBSD), and energy-dispersive X-ray spectroscopy (EDX).

## 2. Materials and Methods

**Figure 3:**
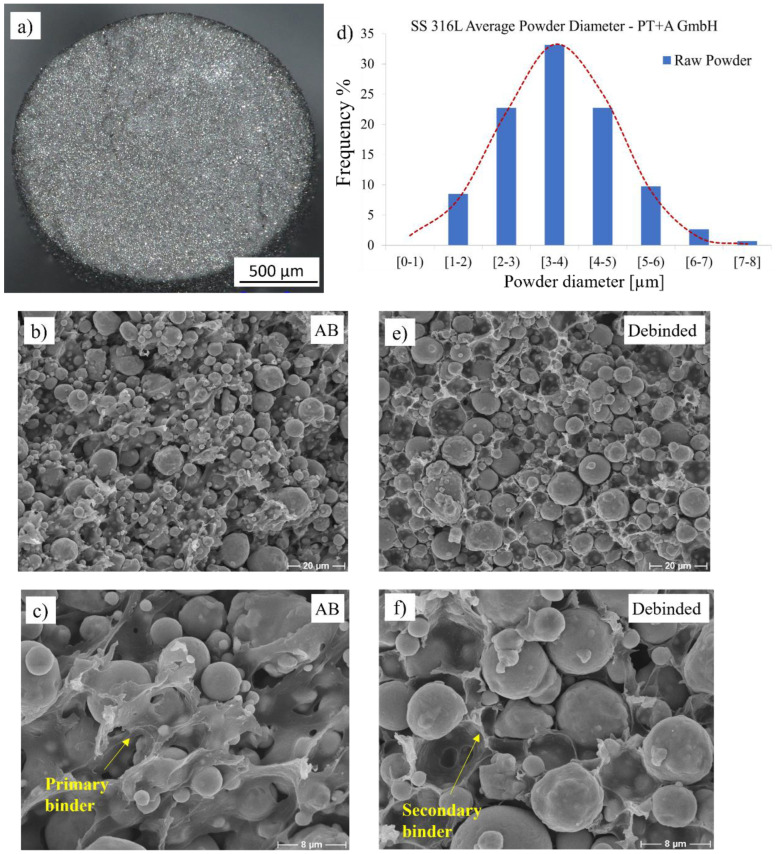
(**a**) Raw filament cross-section at mag. 50×, (**b**) micrograph of feedstock with stainless steel 316L powder embedded by polymeric binder at mag. 1000× and (**c**) mag. 3000×, (**d**) particle size distribution of measured powder in the debinded state, (**e**) micrograph of the debinded geometry at mag. 1000× and (**f**) mag. 3000×.

Cubic geometries with a dimension of 10 × 10 × 5 mm^3^ were shaped using a 1.75-mm-diameter filament acquired from PT+A GmbH (Dresden, Germany) with a 0.4 mm vanadium alloyed high-speed steel nozzle from Slice Engineering (Gainesville, FL, USA). The filament was embedded with micron-sized stainless steel 316 L powder using a polyamide-based backbone, and the chemical composition of the metallic component is provided in Table 1. A cross-sectional micrograph of the raw filament is shown in Figure 3a–c at magnifications of 50×, 1000×, and 3000×, respectively. The average particle size distribution with the diameter of the 316 L powder as $D_{50}$ = 3–4 µm is given in Figure 3d.

**Figure 4:**
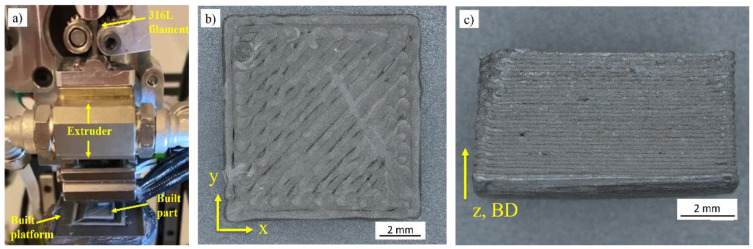
(**a**) Custom in-house FFF machine shaping a geometry, (**b**) XY plane of AB green parts and (**c**) Z-BD plane.

The samples were manufactured using an in-house custom-built FFF machine (Figure 4a), and the as-built (AB) printed geometry is shown in Figure 4b,c in the XY and Z-build direction (BD) planes, respectively. The building parameters, i.e., extruder temperature, bed temperature, layer thickness, and print speed, were 170 °C, room temperature, 150 µm, and 40 mm/s, respectively.

The debinding of the parts was conducted using an acetone solvent with a 99.5% purity acquired from Höfer Chemie (Kleinblittersdorf, Germany) at 45 °C. The sample was considered fully debinded when there was no measurable mass loss of the dissolved binder after 24 h. The sample was removed from the solvent, dried for 12 h, and the mass was recorded using a scale from Kern & Sohn GmbH (Balingen, Germany) until full primary binder removal was achieved in accordance with the filament supplier. A micrograph after the debinding treatment, exhibiting the secondary binder or backbone, is shown in Figure 3e,f at magnifications of 1000× and 3000×, respectively.

### 2.1. Sintering Parameters

**Figure 5:**
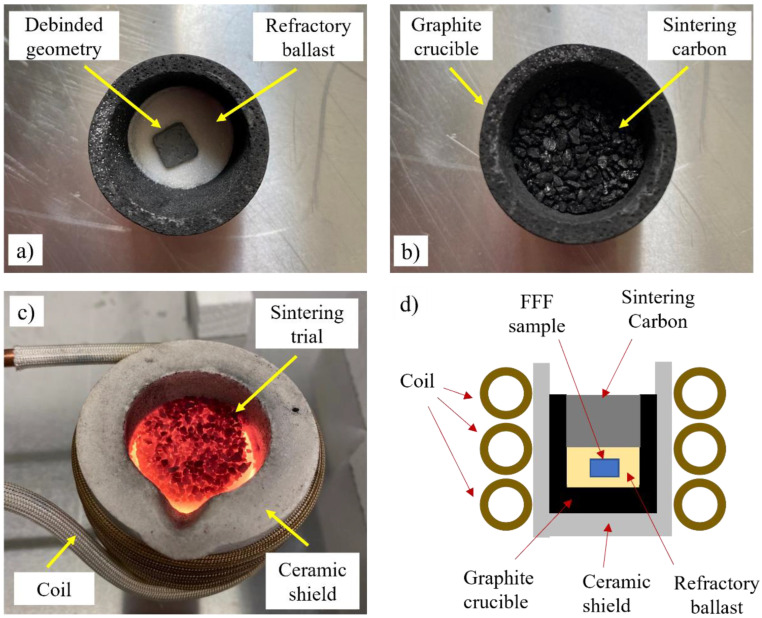
Indirect induction sintering setup with (**a**) the debinded geometry partially buried in the refractory ballast, (**b**) graphite crucible with a top layer of carbon pellets, (**c**) treatment in operation, and (**d**) illustration of the test.

**Figure 6:**
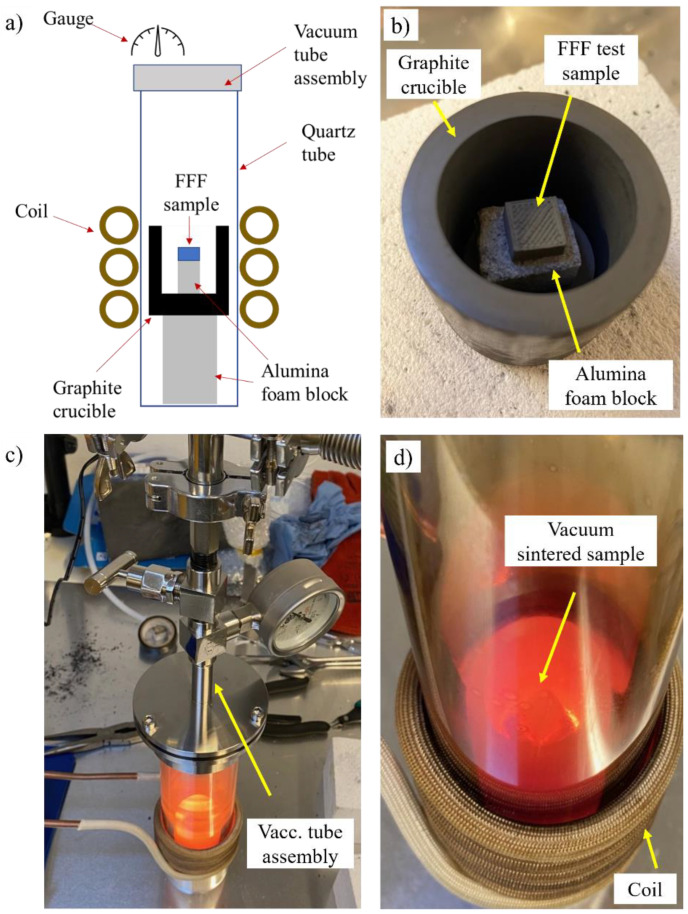
(**a**) Illustration of the test setup used for the indirect induction sintered treatment inside a vacuum tube, (**b**) FFF test sample on top of the alumina foam block surrounded by the graphite crucible, (**c**) vacuum tube assembly during the sintering treatment and (**d**) the sample undergoing a vacuum sintering treatment.

After debinding, the powder geometries were subjected to an indirect induction sintering treatment. The sintering was conducted using a standard 3 kW zero volt switching (ZVS) induction heater operating at a 60 kHz frequency in a decoupled approach, whereby, after shaping and solvent debinding, the parts were prepared for sintering. The sintering temperature was ramped up quickly to 1350 °C and was monitored with the use of a CSmicro 2 MH pyrometer acquired from Optris GmbH (Berlin, Germany). Two different induction sintering methods were conducted to avoid the oxidation of the 316 L powder:

1. The geometries were buried in the Al_2_O_3_ refractory sintering ballast acquired from the Virtual Foundry (Stoughton, WI, USA), with a layer of ~2 cm sintering carbon pellets added on the top layer of the ballast. The test setup is shown in Figure 5, with the debinded geometry partially buried within the refractory ballast (Figure 5a), the carbon pellets and graphite crucible prepared (Figure 5b), the sintering treatment in operation (Figure 5c), and the overall illustration of the experiment depicted (Figure 5d). Different treatments were conducted, whereby the geometries were subjected to soak times of 10 s, 30 s, 1 min, and 4 min. The samples were subsequently allowed to cool to room temperature.

2. Separate sintering trials were performed under vacuum, whereby the debinded specimens were inserted inside a vacuum tube. The geometry was first placed on top of an alumina foam block acquired from MTIxl (Richmond, California, USA), subsequently encased in a high-purity graphite crucible acquired from the LLF Smelting Lab and inserted in the vacuum tube with the vacuum level maintained at 1.8 × 10^−2^ millibar. An illustration of the test setup is provided in Figure 6a, with the test sample on top of the foam block and encased by the crucible, as shown in Figure 6b, the vacuum assembly during operation (Figure 6c), and the sample being subjected to the treatment (Figure 6d). The vacuum sintering trials were conducted for hold times of 10 s, 30 s, 1 min, and 6 min, after which the specimens were left to cool to room temperature.

### 2.2. Microstructure and Atomic Composition Measurements

The sintered specimens were prepared for microstructural microscopy through grinding and polishing. Optical micrograph measurements were performed using an Axioscope 7 (Zeiss, Oberkochen, Germany) at magnifications of 50× and 500×. The images were stitched together with the multistage functionality of the ZEN core software (Zeiss, Oberkochen, Germany), and the post-processing of the average density was determined from the micrographs with Fiji Image-J analysis software. Three different specimens per condition were used for the overall density calculations. To further characterize the overall average porosity diameter and aspect ratio, the diameters of 40 porosities per condition were measured in the refractory and vacuum-sintered specimens. Additional surface topography measurements were performed at a magnification of 200× using a VHX-7000 digital microscope (Keyence GmbH, Neu-Isenburg, Germany). The results were analyzed using the 3D measurement functionality of the VHX software, and the average surface roughness was quantified via the surface topography measurements using the Fiji Image-J surface assessment package.

Electron Backscatter Diffraction (EBS) maps were generated using a Hitachi S-2700 scanning electron microscope (SEM, Japan) at a magnification of 3000×. The results were analyzed using Channel 5 software from Oxford instrument. Energy-dispersive X-ray spectroscopy (EDX) measurements and wavelength-dispersive X-ray spectroscopy (WDX) elemental mapping were undertaken using a JEOL Field Emission Electron Probe Microanalyzer (FE-EPMA) JXA-8530F microprobe using the JEOL EDX and WDX system, conducted at a mag. of ×12,000 at 15 kV.

## 3. Results & Discussion

### 3.1. Sintering Kinetics & Porosities

**Figure 7:**
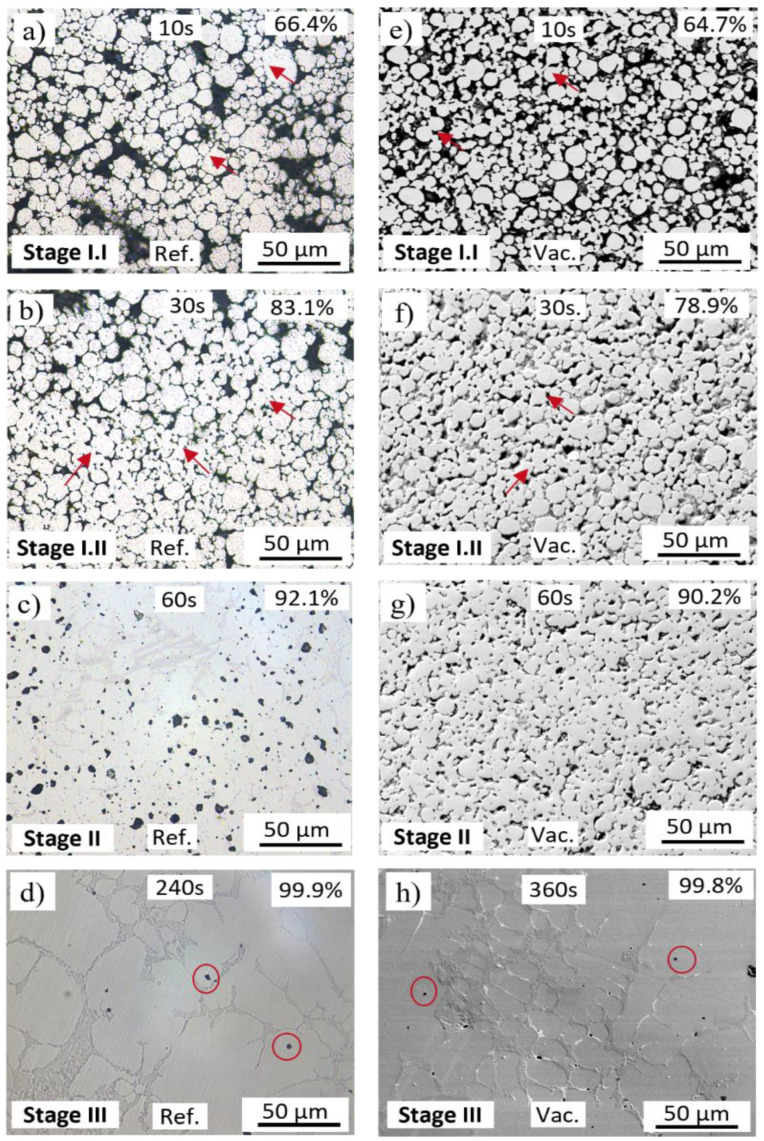
Micrographs of the indirect induction sintered FFF 316L stainless steel in the refractory ballast after (**a**) 10 s, (**b**) 30 s, (**c**) 60 s, and (**d**) 240 s, and sintered in the vacuum tube after (**e**) 10 s, (**f**) 30 s, (**g**) 60 s, and (**h**) 360 s.

Micrographs of the indirect induction-sintered geometries buried in the refractory ballast after a treatment of 10 s, 30 s, 1 min, and 4 min are shown in Figure 7a–d, respectively, with the samples sintered under a vacuum for soak times of 10 s, 30 s, 1 min, and 6 min provided in Figure 7e–h. The red arrows in Figure 7a,b,f,g, illustrate example locations in which the neck growth between the particles in direct contact begins to coalesce, whereas the red circles in Figure 7d,h depict isolated micropores in the final stages of sintering.

The average densities of the refractory ballast sintered samples after 10 s, 30 s, 1 min, and 4 min is 66.4%, 83.1%, 92.1%, and 99.9%, respectively, whereas the samples sintered in a vacuum after 10 s, 30 s, 1 min, and 6 min are 64.7%, 78.9%, 90.2%, and 99.8%, respectively. The driving force to densify the powder compacts at the sintering temperatures is the reduced surface energy caused by the formation of intergranular bonds, reducing the surface area [35,49]. Under both conditions, the specimens follow the same kinetics as other sintering strategies conducted on powder metallurgy, consisting of three clearly identified stages. In Figure 7a,b,e,f, neck growth is triggered, whereby particles in direct contact form grain boundaries through atomic diffusion and necks begin to form at the contact points (stage I). Surface diffusion is the dominant mass-transport mechanism at this stage [50]. In Figure 7c,g, the intermediate sintering stage is characterized by the densification and grain growth of the specimens, with pores being isolated and closed (stage II). Grain boundary diffusion dominates at this stage [50], causing the material to migrate from the core of the powder particles to the surface, resulting in densification. Lastly, Figure 7d,e showcase the final sintering stage (stage III). Once most pores are closed, and as the grain boundaries increase, the gas trapped within the pores escapes to the surface, with the subsequent effect of decreasing the volume of the sintered part.

**Figure 8:**
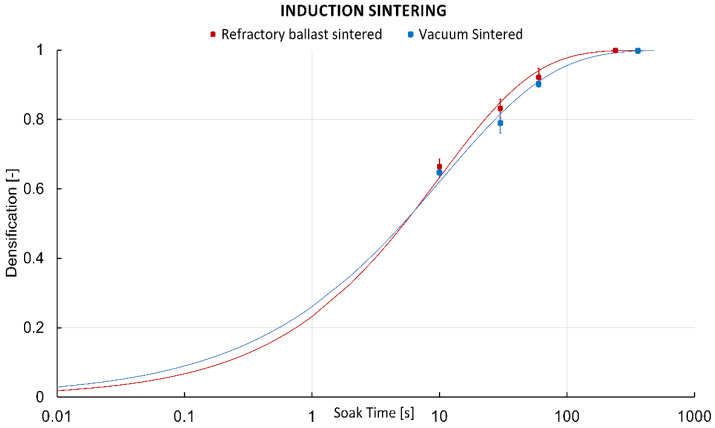
Johnson–Mehl–Avrami–Kolmogorov (JMAK) model curves of the 316L FFF parts sintered within the refractory ballast (red), and in a vacuum (blue).

The Johnson–Mehl–Avrami–Kolmogorov (JMAK) model for diffusion-controlled growth was used to describe the kinetics of densification for both conditions (Figure 8). The densities of the samples sintered within the refractory ballast are depicted in red, whereas the samples sintered under a vacuum are depicted in blue. Data points, including the standard deviation, are represented by thick colored markers, with the model fitted as a curve. For the model, it was assumed that nucleation occurs homogeneously and randomly throughout the full volume of the specimens, and growth occurred at the same rate in all vectors (i.e., particles grow into spheres). Additionally, the model assumes that nucleation begins from point spheres. To fit the model, the Avrami relationship (Equation (1)) was used, with *n* and *K* found after plotting the relationship in Equation (2), whereby the *ln[−ln(1−Y(t))]* against time revealed *n* and *K* as the slope and intercept values, respectively. Here, *n* is the Avrami exponent, *K* is the nucleation rate, and *Y(t)* is the extent of the transformation at time *t(s),* with *n* and *K* describing the nucleation and growth of the particles. The results of the Avrami exponent and nucleation rate presented in Table 2 suggest that there is a decreasing nucleation rate [51], with the general Avrami exponent equation characterized by Equation (3), whereby *a* is the nucleation index with 0 < *a* < 1 for decreasing nucleation rates, *b* is the growth dimensionality (1, 2, or 3), and *c* the index of growth with *c* = 0.5 for diffusion-controlled growth [51]. The conclusions indicate that FFF powder geometries sintered via the indirect induction route can be adequately predicted using the JMAK model.

(1) $$Y=1-e^{-Kt^{n}},$$

(2) ln(−ln[1−Y(t)])=lnK+nlnt,

(3) n=a+bc,

The refractory ballast sintered specimens exhibit a faster densification rate as opposed to the specimens sintered under a vacuum. This is ascribed to the differing heat-transfer mechanisms of the process, with the refractory samples being subjected to the conducting heat of the ballast, which is in direct contact with the graphite crucible, whereas the vacuum-sintered specimen is through pure radiation, which has a reduced effective heat due to the loss of free space. In a vacuum, the samples closer to the crucible will experience higher effective heat compared to those at the center of the crucible, but with the detrimental effect of having an uneven distribution of radiated heat along the sintered part if not symmetric along the crucible principal geometric axes. However, high densities of 99.9% and 99.8% are reached rapidly after 4 min and 6 min in the refractory ballast and vacuum-sintered specimens, respectively, being a notable improvement compared to the conventional furnace approach, which reached between 2 h and 13 h heating cycles in recent studies of sintered FFF stainless steel 316L [52,53,54,55].

**Figure 9:**
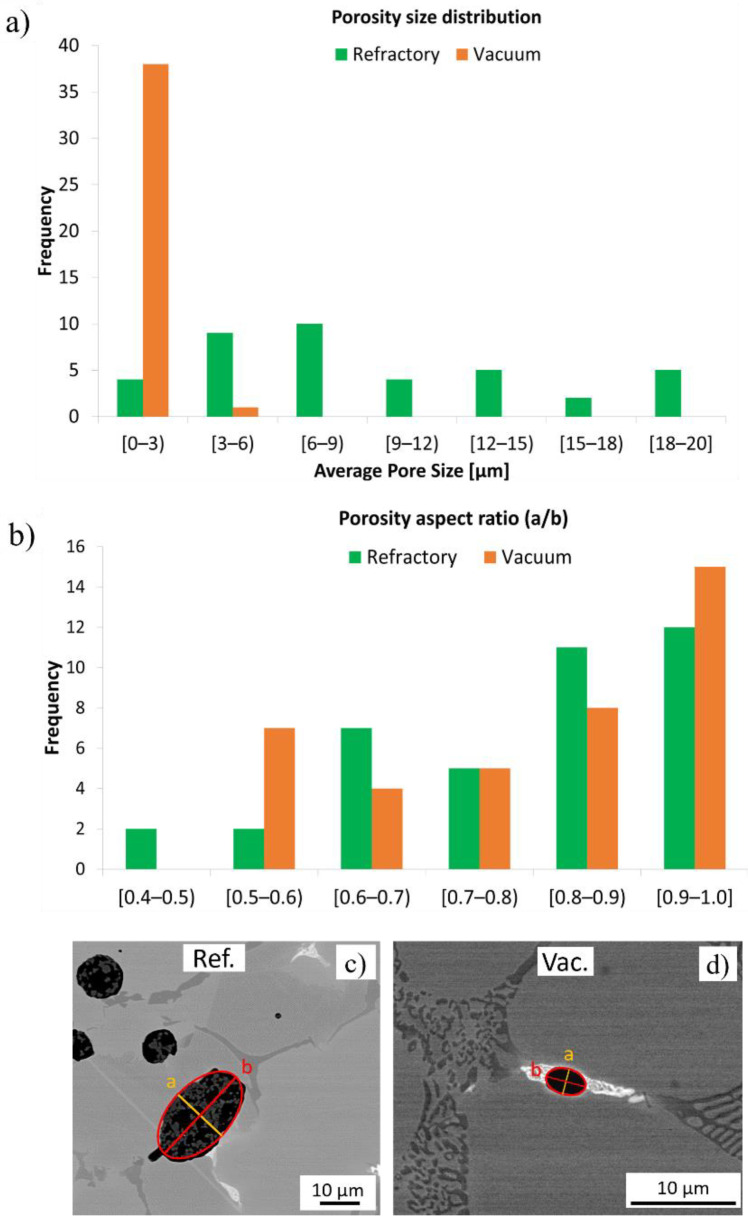
(**a**) Mean porosity size as a function of frequency and (**b**) porosity aspect ratio for the refractory-sintered (green) and vacuum-sintered (orange) specimens. Subfigures (**c**,**d**) show the shape of the porosities for the refractory ballast and vacuum sintered, respectively, with the measured semi-minor axis, a, and semi-major axis, b, indicated within the micrographs.

The average porosity measurements for the refractory ballast after 240 s and the vacuum-sintered specimens after 360 s are provided in Figure 9a. The overall average porosity size is 9.18 ± 5.27 µm and 1.75 ± 0.87 µm for the refractory ballast and vacuum sintered, respectively. In the vacuum-sintered condition, the average porosity size is considerably smaller than those sintered within the refractory ballast, exhibiting a decrease in porosity size of 80.92%. This can be attributed to the longer soak time compared to the refractory-sintered specimens. Figure 9b plots the ellipticity of the porosities, with example Figure 9c,d showing magnified representative porosities for the refractory ballast and vacuum sintered, respectively, including a fitted ellipse of semi-minor axis a and semi-major axis b. The values close to 1 in the ratio between a/b represent spherical micropores, whereas the values approaching 0 describe highly elliptical pores. It is apparent that there is a trend for pores to become more spherical as the soak time of the sintered specimens increases. There is a detrimental influence of the micropores as there is a stress concentration around these voids that act as nucleation sites for crack initiation and propagation, in particular near the surface zone [56]. Consequently, having a reduced pore size has a positive impact on increasing the fatigue strength and lifetime of load-bearing parts [57].

### 3.2. Microstructure

**Figure 10:**
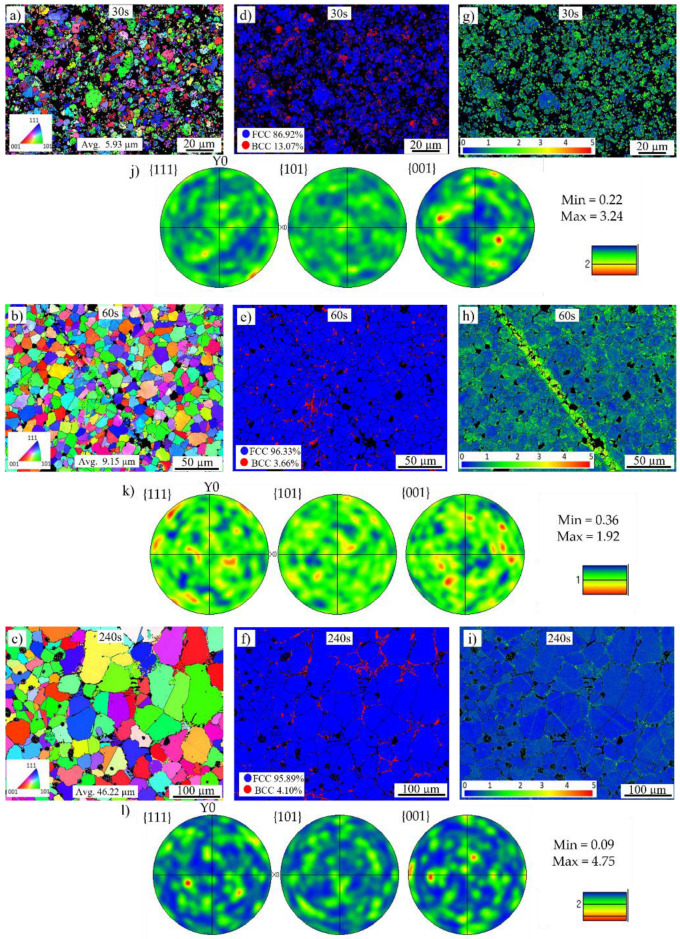
EBSD maps of the refractory ballast sintered specimens after (**a**) 30 s, (**b**) 60 s, (**c**) 240 s, phase maps of the specimens after (**d**) 30 s, (**e**) 60 s, (**f**) 240 s, local misorientation maps after (**g**) 30 s, (**h**) 60 s, (**i**) 240 s and Euler pole figures after (**j**) 30 s, (**k**) 60 s, and (**l**) 240 s.

**Figure 11:**
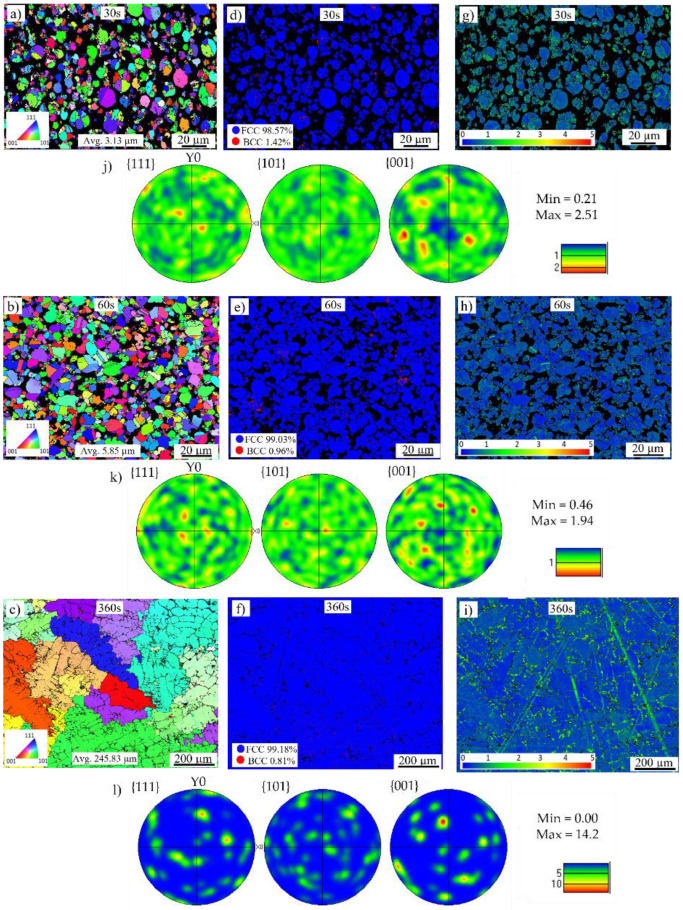
EBSD maps of the vacuum sintered specimens after (**a**) 30 s, (**b**) 60 s, and (**c**) 360 s, phase maps of the specimens after (**d**) 30 s, (**e**) 60 s, (**f**) 360 s, local misorientation maps after (**g**) 30 s, (**h**) 60 s, (**i**) 360 s and Euler pole figures after (**j**) 30 s, (**k**) 60 s, and (**l**) 360 s.

The EBSD microstructures of the refractory ballast sintered specimens after soak times of 30 s, 60 s, and 240 s, and the vacuum-sintered specimens after soak times of 30 s, 60 s, and 360 s, are provided in Figure 10a–l and Figure 11a–l, respectively, with non-indexed points due to the porosities shown in black. The inverse pole figure maps in the normal direction (IPFz) are shown in Figure 10a–c and Figure 11a–c. The microstructures of the refractory ballast sintered are completely equiaxed, evolving from a fine average grain size of 5.93 µm and 9.15 µm to 46.22 µm after 30 s, 60 s, and 240 s. In the vacuum-sintered case, the grains evolve from an average grain size of 3.13 µm and 5.85 µm to 245.83 µm after 30 s, 60 s, and 360 s. As expected, the grain size under both conditions increases gradually over time, which appears during stages II and III of powder sintering [49,50]. The decrease in the surface area between the fine-grained and larger-grain material, with the subsequent effect of decreasing the grain-boundary energy, renders a free-energy change that results in grain growth [58]. After 240 s, the microstructure sintered within the refractory ballast exhibits a similar final grain size, as with the work shown in [59], which reached ~45 µm after a 180 min sintering time on sintered stainless steel 316 L shaped using FFF. Several trapped pores are found inside the equiaxed grains after 240 s of the refractory-sintered samples, which is typical during the last stage of sintering [49]. Annealing twins are additionally identified during the grain growth of the vacuum-sintered specimens after 60 s, which has been reported previously during the grain growth of powder sintering FFF stainless steel 316 L [52,60]. Concerning the vacuum-sintered specimens, large dendritic grains are observed after 360 s, which is connected to the extended soak time compared to the refractory ballast sintered. The dendritic morphology at the end of the vacuum-sintered specimens is attributed to the location in which EBSD maps have been captured, which was performed near the surface zone at the sample–block interface within this condition.

The Euler pole figures in the {001}, {101}, and {111} planes are given in Figure 10j–l and Figure 11j–l. The figures depict the texture defining the orientation of the crystals within the solid sintered mass. Initially the texture of the refractory-sintered sample is weak, with ~3.24 and 1.92 times random intensity after 30 s and 60 s, respectively, presenting a final isotropic randomized crystallographic texture with a maximum intensity of ~4.75 times after 240 s of soak time. For the vacuum-sintered specimens, the texture exhibits a random intensity in the {001} plane with ~2.51 and 1.94 times after 30 s and 60 s, respectively, presenting a strong texture with a maxima of ~14.2 times the uniform intensity after a sintering time of 360 s. The larger texture in the vacuum-sintered condition after 360 s is linked to the configuration of the sample during the treatment, which was located on top of the ceramic foam block, with the EBSD map captured close to the sample–block interface. In this configuration, there is a detectable directional solidification with a preferred growth along the <001> direction close to the ceramic block. It has been reported that in cubic austenite face-center cubic (FCC) stainless steel 316L with dendrite crystals, having a fast growth parallel to the heat flow, <001> is the preferential solidification direction [61,62]. Despite the larger texture on the vacuum-sintered specimen, the microstructure exhibits a higher isotropy compared to stainless steel 316L processed through powder bed fusion [63,64].

Phase maps are provided in Figure 10d–f and Figure 11d–f, with blue representing the γ-austenite FCC crystalline structure and red depicting the δ-ferrite body-center cubic (BCC) structure. The quantified values of the austenite and ferrite phases are given in Table 3. The austenitic γ FCC microstructure is the dominant phase in both sintered conditions, co-existing with a small proportion of the δ-ferrite phase along the boundary of the powder granules. A similar phase distribution was reported in FFF stainless steel 316L sintered using the conventional furnace approach [65], with the presence of the δ-ferrite phase being attributed to the preparation of the powder feedstock [66].

**Figure 12:**
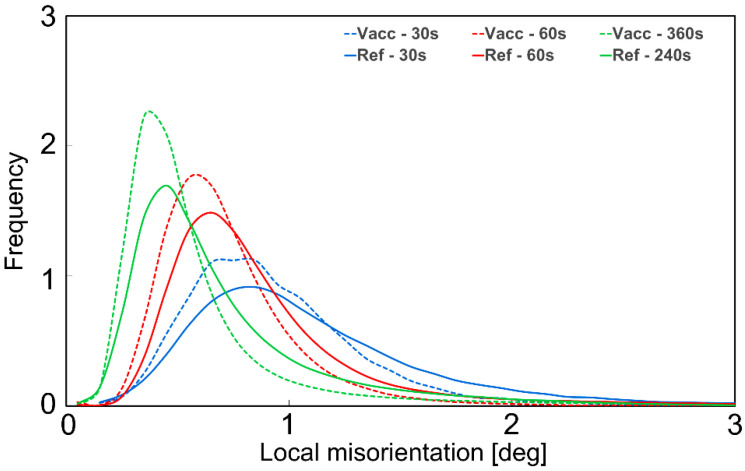
Local misorientation distributions of the refractory ballast (solid lines) and vacuum-sintered (dashed lines) samples.

Local crystallographic Kernel Average Misorientation (KAM) maps underlining regions of higher deformation are given in Figure 10g–i and Figure 11g–i with a scale between 0° and 5°. These maps measure the average misorientations between neighboring pixels, looking at regions extending up to their seventh nearest neighbor, excluding misorientations beyond 5°. The results are mapped in Figure 12, with the solid curves representing the refractory ballast sintered and the dashed lines representing the vacuum sintered, illustrating the evolution of the misorientation levels throughout the sintering treatments. In both conditions, the samples display a higher crystallographic misorientation, which is initially stored in the feedstock powder, indicating the presence of a high initial dislocation density [67]. As the soak time increases, the misorientation levels are reduced due to the annealing of dislocations, which is typical of heat treatments conducted on stainless steel 316 L produced through additive manufacturing [68]. The samples sintered within the refractory ballast display a higher misorientation after 240 s compared to the vacuum-sintered samples after 360 s, which is connected to the difference in sintering time.

### 3.3. Composition Analysis

**Figure 13:**
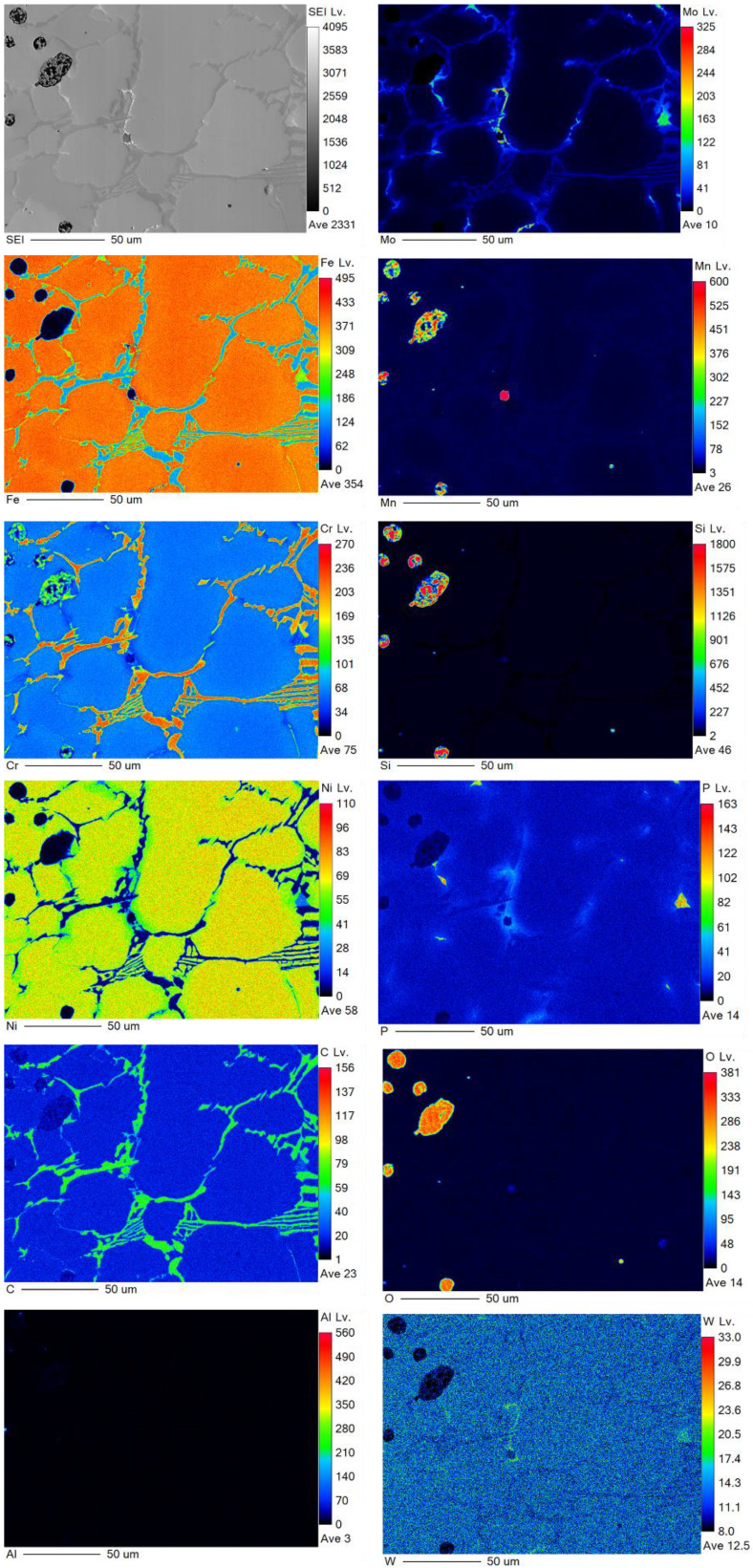
WDX measurement for the refractory ballast sintered for 240 s, mapping Fe, Cr, Ni, Mo, Mn, Si, P, C, O, Al, and W.

**Figure 14:**
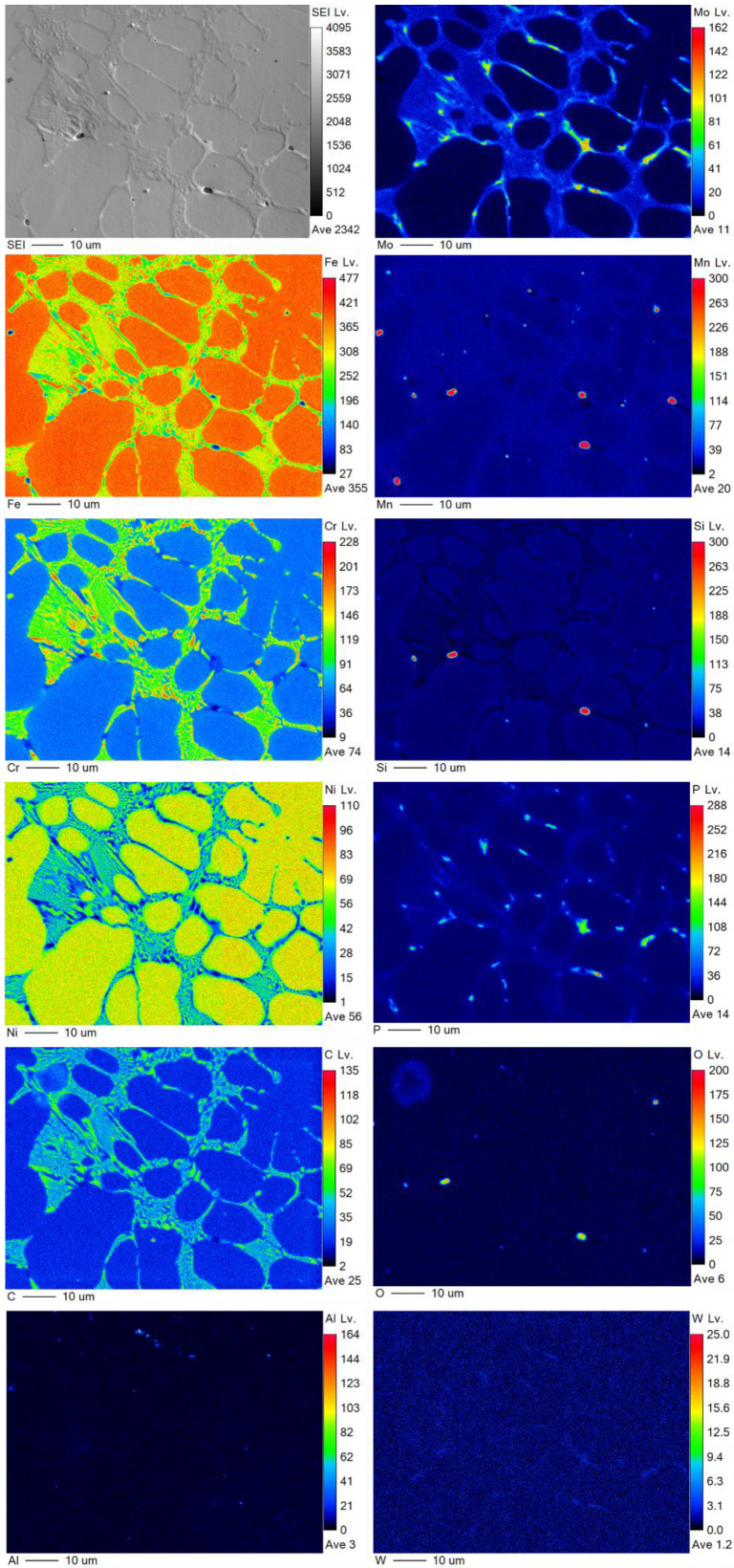
WDX measurement for the vacuum-sintered for 360 s, mapping Fe, Cr, Ni, Mo, Mn, Si, P, C, O, Al, and W.

Elemental wavelength-dispersive X-ray (WDX) measurements for the chemical mapping of the refractory-sintered samples after 240 s, and vacuum-sintered samples after 360 s, are provided in Figure 13 and Figure 14, respectively. The mapped elements include Fe, Cr, Ni, Mo, Mn, Si, P, C, O, Al, and W. In both conditions, there is an apparent carbonization with higher concentrations of carbon (C) around the grain boundaries. These measurements, coupled with an observed enrichment of chromium (Cr) and molybdenum (Mo) around the grain boundaries in both conditions, indicate chromium-rich carbide precipitates at the grain boundaries, which is congruent with similar studies on sintered stainless steel 316L [54,69]. In these studies, they justified that since carbon diffusion and carbide precipitation are faster than chromium diffusion, there is a formation of chromium-rich carbides, which can result in chromium depletion along the boundaries. This can have a negative impact on the material properties, resulting in reduced elongation and embrittlement [54].

In the refractory-sintered specimens, an uneven distribution of impurities of oxygen (O) and the enrichment of silicon (Si) and manganese (Mn) co-exist, trapped within the pores. Oxygen levels are significantly reduced when sintering under a vacuum atmosphere, although higher vacuum levels are suggested in future work, as there is an apparent small absorption of oxygen around the micropores in the vacuum-sintered specimens.

**Figure 15:**
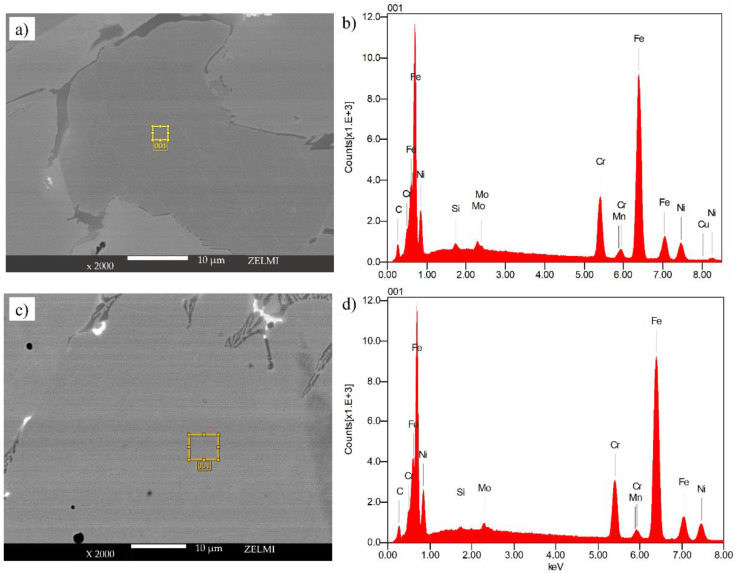
EDX measurement sites for samples sintered in (**a**) refractory ballast for 240 s, (**c**) vacuum for 360 s, with (**b**) the chemical composition of the refractory ballast and (**d**) vacuum-sintered samples.

Detailed quantitative results of the energy-dispersive X-ray (EDX) composition analysis within the bulk of the grains for the samples sintered within the refractory ballast and vacuum after 240 s and 360 s, respectively, are provided in Figure 15a,c, with the element-composition counts mapped in Figure 15b,d. Quantified values in wt. % are provided in Table 4 for both sintering conditions. Compared to DIN 17440 stainless steel 316L, both of the sintered specimens have endured significant carbonization, which is associated with the small proportions of carbon residues from the organic binder mass [70], coupled with residues that are evaporated from the graphite crucible during the sintering treatment. To mitigate carbonization, it is advisable to study the effect of different crucibles on the chemical composition of high-density induction-sintered stainless steel 316 L.

**Figure 16:**
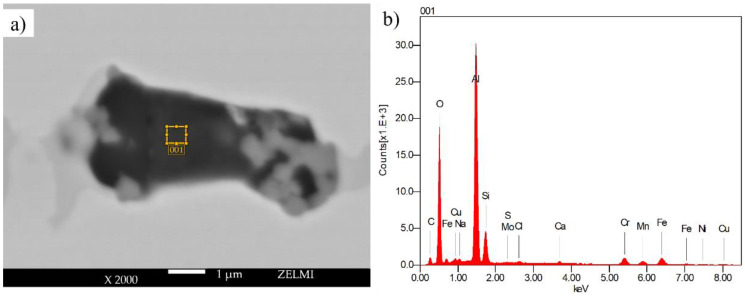
EDX measurement within a void in the sample sintered in (**a**) refractory ballast for 240 s and (**b**) the chemical composition of the refractory of the refractory ballast sintered.

At higher magnifications, impurities of aluminum and oxygen were detected within the voids on the sample sintered inside the refractory ballast, with the measured location shown in Figure 16a and the quantified chemical composition exhibited in Figure 16b. An increased aluminum and oxide concentration is measured within the pores, with the quantified values given in Table 5, which is attributed to the trapped refractory Al2O3 ballast within the near-surface pores.

### 3.4. Shrinkage and Surface Quality

**Figure 17:**
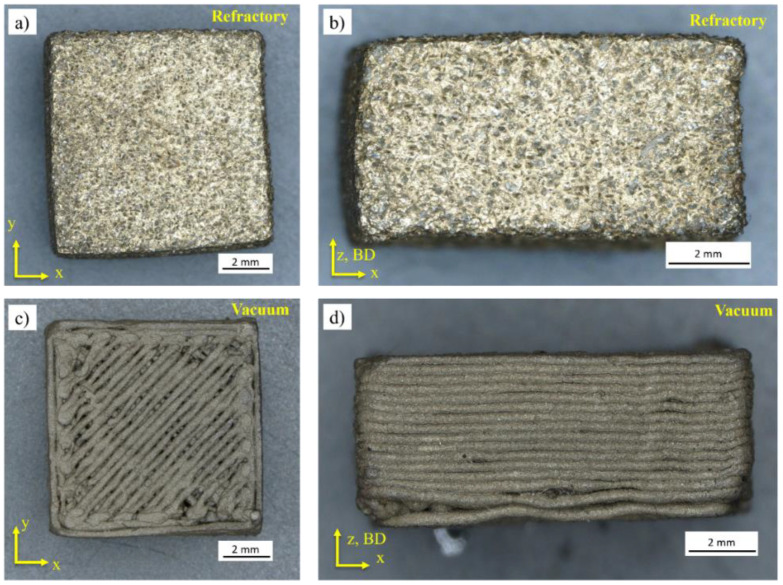
Indirect induction sintered specimens within the refractory ballast for a soak time of 240 s in the (**a**) XY and (**b**) ZX planes, and vacuum sintered for a soak time of 360 s in the (**c**) XY and (**d**) XZ planes.

High-density as-sintered specimens treated within the refractory ballast and under a vacuum are shown in Figure 17a–d in the XY and XZ planes, respectively. Under both test conditions, there is a measurable shrinkage compared to the debinded state, with an average resulting length of 8.53 ± 0.83 mm, 8.51 ± 1.28 mm, and 4.66 ± 0.26 mm in the X, Y, and Z directions for the refractory-sintered samples, and 8.91 ± 0.17 mm, 8.81 ± 0.11 mm, and 4.68 ± 0.35 mm in the X, Y, and Z directions for the vacuum-sintered samples. In contrast to the original specimens, this represents a shrinkage of 14.61%, 14.81%, and 6.65% in the X, Y, and Z for the refractory ballast sintered, and of 10.88%, 11.94%, and 6.27% in the X, Y, and Z for the vacuum-sintered samples. The results fit within the filament supplier guidelines that caution for an overall shrinkage of ~15%. Shrinkage upon sintering is a well-known effect of powder-based metallic additive manufacturing and has been extensively reported in relation to stainless steel 316L produced through FFF [71,72,73]. The effect is attributed to the bulk transport mechanism appearing during the final stages of sintering, whereby neck growth and the elimination of pores are dominated by the atomic particles that migrate from the bulk of the neighboring granules at the contact points [74]. The mitigation of the shrinkage effect is traditionally addressed by increasing the powder packing density of the filament feedstock (i.e., infill density), coupled with an over-dimensioning of the geometry to compensate for the shrinkage behavior [75]. However, for the vacuum-sintered samples, a slightly lower shrinkage and a standard deviation are found as opposed to the refractory-sintered condition. The fast and clean heating due to the concentration of high power densities during an indirect induction treatment inside a vacuum enables the precise control of the shrinkage behavior while ensuring high-density parts inside the bulk. On the other hand, it is observed that the fast heating rates have caused a shrinkage of individual deposited tracks on the top outside layer under this condition (Figure 15c), suggesting that an increase in the AB infill density coupled with tailored heating rates needs to be further studied for this sintering method.

**Figure 18:**
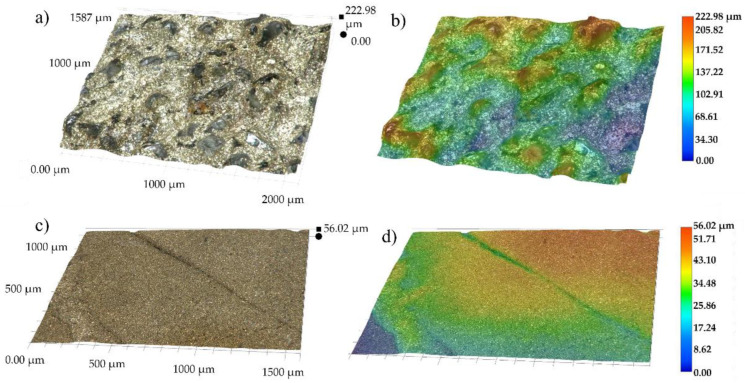
3D surface topography measurements at mag. 200× for specimens sintered within (**a**) the refractory ballast with (**b**) color-coded, and (**c**) vacuum-sintered with (**d**) color-coded.

Three-dimensional surface topography micrographs at mag. 200× for the as-sintered refractory ballast and vacuum-sintered specimens are provided in Figure 18a–d, with the surface shape and roughness quantified via color codes in Figure 18b,d. The deviation of the sintered surfaces from the mean plane in the refractory-sintered and vacuum-sintered conditions is 111.49 µm and 28.01 µm, respectively. The average surface roughness (Ra) for the refractory-sintered and vacuum-sintered specimens is 41.37 µm and 23.44 µm, respectively. In the refractory-sintered case, the surface displays a high roughness with clumps of Al_2_O_3_ refractory fused to the surface. Post-processing in this condition is necessary to improve surface roughness. In the vacuum-sintered specimen, the surface exhibits a clean and smooth progression, with only local deviations found between the filament deposition tracks. A lower surface roughness with a percentage decrease of 43.32% is observed in this state compared to the refractory-sintered samples. Vacuum sintering without refractory ballast can be a favorable alternative to render near-net shape parts without additional surface-improvement post-processing steps.

## 4. Conclusions

Indirect induction sintering of stainless steel 316 L shaped through FFF was shown in this work, revealing that high densities can be achieved efficiently in very short sintering cycles. The following conclusions are made:

1. The capacity to sinter under vacuum conditions via pure thermal radiation can achieve bulk densities of up to 99.8% in only 6 min of soak time and densities of 99.9% in 4 min when sintered within a refractory ballast under an open atmosphere. The highly concentrated power densities of induction sintering result in the technology being highly efficient. The reported improvements in the sintering kinetics exceed those obtained with traditional state-of-the-art methods and underline a new significant advantage of combining local sintering during the manufacturing process, cutting the processing times of FFF parts. However, the sintering time depends on the volume of the geometry, whereby higher soaking times will be necessary for larger bodies.
2. The average porosity size and ellipticity of sintered FFF parts can be reduced by increasing the soak time. When sintering is conducted under a vacuum for 120 s increased soak time, the average porosity size can be reduced by 80.92%.
3. By tailoring the heating rates and transfer mechanism of the process, an isotropic FCC microstructure is possible. It is advisable that dedicated sintering profiles are developed in future work to ensure high densities while rendering small equiaxed, fine grains.
4. The careful extraction of the fumes generated from the heating of a graphite crucible should be considered to mitigate the absorption of carbon residues by the metallic solid mass. Further studies should focus on using other types of crucibles.
5. An improved surface roughness with a decrease of 43.32% can be achieved on samples sintered under a vacuum compared to the refractory ballast sintered samples. The correlation between the surface roughness and the shrinkage of dense sintered parts needs to be further investigated in future work.

A hybrid machine combining local indirect induction sintering with FFF AM was proposed, allowing to sinter parts locally without the need for part transportation to separate furnaces. In situ sintering is a promising novel technique to process material extrusion additively manufactured metallic parts embedded with powdered granules, reducing the costs and health risks inherent to other traditional approaches.

## 5. Patents

The combination of material extrusion AM with local sintering is a novel hybrid additive manufacturing method with a patent application filed by the Technische Universität Berlin [33].

## Figures and Tables

**Table 1:** Chemical composition of 316L stainless steel acquired from PT+A GmbH, wt. %.

	Cr	Ni	Mo	Mn	Si	P	C	Fe
316L filament	17.0	10.2	2.2	1.58	0.8	0.03	0.018	Balance

**Table 2:** Resulting n and K values used for the JMAK model.

	Avrami Exponent (n)	Nucleation Rate (K)
Refractory ballast sintered	0.5788225	0.2642992
Vacuum sintered	0.5063679	0.3019814

**Table 3:** Quantified percentage values of the austenite and ferrite phases for the refractory-sintered after 30 s, 60 s, and 240 s, and the vacuum-sintered after 30 s, 60 s, and 240 s.

	Austenitic γ	Ferrite δ
Refractory (30 s)	86.92%	13.07%
Refractory (60 s)	96.33%	3.66%
Refractory (240 s)	95.89%	4.10%
Vacuum (30 s)	98.57%	1.42%
Vacuum (60 s)	99.03%	0.96%
Vacuum (360 s)	99.18%	0.81%

**Table 4:** Chemical composition of geometries sintered in the refractory ballast and vacuum, wt. %.

	Cr	Ni	Mo	Mn	Si	C	Fe
Refractory (240 s)	13.13	11.09	1.29	0.38	0.30	2.87	Balance
Vacuum (360 s)	12.69	11.63	1.23	0.52	0.14	2.73	Balance
DIN 17440	16.0–18.0	10.0–14.0	2.0–3.0	0.0–2.0	0.0–1.0	0.0–0.03	Balance

**Table 5:** Wt. % of the chemical composition within the pores of refractory ballast sintered.

	Cr	Ni	Mo	Mn	Si	C	O	Al	Fe
Refractory (240 s), voids	4.82	0.91	0.17	2.34	5.52	6.18	40.73	28.42	Balance

## Data Availability

The data presented in this study are available upon request from the corresponding author. The data are not publicly available due to restrictions related to the ongoing patent evaluation procedures.
